# The Role of the Membrane in the Structure and Biophysical Robustness of the Dengue Virion Envelope

**DOI:** 10.1016/j.str.2015.12.011

**Published:** 2016-03-01

**Authors:** Tyler Reddy, Mark S.P. Sansom

**Affiliations:** 1 Department of Biochemistry, University of Oxford, South Parks Road, Oxford OX1 3QU, UK

**Keywords:** viral envelope, lipid bilayer, structure and dynamics, diffusion, dengue virus

## Abstract

The dengue virion is surrounded by an envelope of membrane proteins surrounding a lipid bilayer. We have combined the cryoelectron microscopy structures of the membrane proteins (PDB: 3J27) with a lipid bilayer whose composition is based on lipidomics data for insect cell membranes, to obtain a near-atomic resolution computational model of the envelope of the dengue virion. A coarse-grained molecular dynamics simulation on the microsecond timescale enables analysis of key biophysical properties of the dengue outer envelope. Properties analyzed include area per lipid values (for a spherical virion with a mixed lipid composition), bilayer thickness, and lipid diffusion coefficients. Despite the absence of cholesterol from the lipid bilayer, the virion exhibits biophysical robustness (slow lipid diffusion alongside stable bilayer thickness, virion diameter, and shape) that matches the cholesterol-rich membrane of influenza A, with similarly anomalous diffusion of lipids. Biophysical robustness of the envelope may confer resilience to environmental perturbations.

## Highlights

• The dengue virus envelope is a lipid bilayer plus an outer layer of membrane proteins
• The structures of the proteins plus lipidomics data were used to model the envelope
• Microsecond MD simulations revealed the dynamic behavior of the lipid bilayer
• Protein interactions confer “raft-like” robustness on a cholesterol-free membrane

## Graphical Abstract

**Figure:**
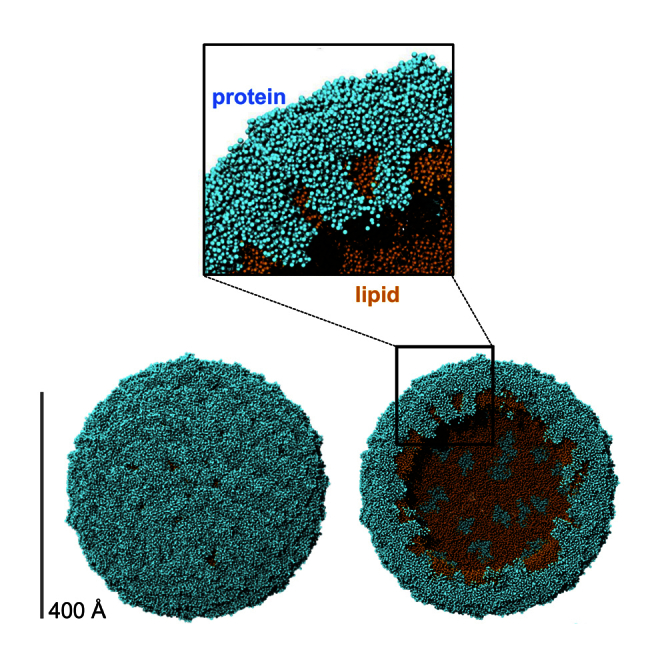

## Teaser

Reddy and Sansom have used coarse-grained MD simulations to model the complete envelope of a dengue virion, including the lipid bilayer. Microsecond MD simulations revealed the dynamic behavior of the lipid bilayer, showing that extensive interactions of the lipids with envelope proteins confer “raft-like” robustness on a cholesterol-free membrane.

## Introduction

Dengue virus is an enveloped flavivirus carried by mosquitos, and is a major health burden (Bhatt et al., 2013). Dengue was the second human disease known to be caused by a virus (Henchal and Putnak, 1990), and dengue virus is a re-emerging pathogen (Morens and Fauci, 2008). There is no specific treatment or approved vaccine, and the *Aedes aegypti* (and *Aedes albopictus*) mosquito vectors are spreading, being linked to global warming and urbanization (Gubler, 2002). Furthermore, there are four circulating dengue virus serotypes, and prior infection with a single serotype can increase the likelihood of severe complications following infection with another serotype, resulting in the challenge of developing a tetravalent vaccine (WHO Fact Sheet 117). Consequently, there is a need to improve our understanding of the molecular structure and biophysical properties of dengue virus to aid the development of novel therapies.

Enveloped viruses have an outer membrane which contains viral membrane proteins, plus lipids derived from the host cell membranes (Ivanova et al., 2015, Polozov et al., 2008). Thus the lipids of the envelope form a key structural component of the virion (Perera et al., 2012). More generally, lipids and the life cycles of many viruses are inextricably linked (Heaton and Randall, 2011). The viral membrane proteins play key roles in enveloped viruses such as dengue, for example in enabling viral entry into host cells (Zhang et al., 2013a), as fusion of the membrane of the virion and the host cell is essential for an enveloped virus to infect a cell (Harrison, 2015).

The dengue virus structure consists of a 50-nm diameter particle with a lipid envelope, and two types of membrane protein, M and E (recently reviewed by, e.g., Cruz-Oliveira et al., 2015). Cryoelectron microscopy (cryo-EM) studies of the dengue virion at 3.5 Å resolution have revealed the structures of the two envelope membrane proteins (Fibriansah et al., 2015, Zhang et al., 2013a) and have provided a clear indication of the location of the lipid bilayer relative to these proteins in the envelope, but the detailed structure of the bilayer remains unresolved. For an understanding of the biophysical stability of dengue virus, it is important to characterize the in situ structure and dynamics of the lipid bilayer component of the envelope. This is especially relevant as lipid envelopes provide exceptional biophysical stability to viruses, for example enabling influenza A to survive for >3 years in distilled water (Stallknecht et al., 1990). Furthermore, alphaviruses, which are classified into group IV along with dengue, have a similar structure, with an outer lipid envelope covered by serologically reactive glycoproteins (Knipe and Howley, 2015). Thus, insights into the structural properties of the dengue virion may be transferable to medically relevant alphaviruses (e.g., Chikungunya virus, Venezuelan equine encephalitis virus).

Molecular dynamics (MD) simulations have previously enabled modeling of the structure and dynamics of several enveloped virions, including influenza A (Durrant and Amaro, 2014, Reddy et al., 2015) and HIV-1 (Ayton and Voth, 2010). Non-enveloped virions including satellite tobacco mosaic virus (Freddolino et al., 2006) and rabbit hemorrhagic disease virus (Wang et al., 2013), and the viral capsids of HIV-1 (Zhao et al., 2013) and Rous sarcoma virus (Goh et al., 2015) have also been studied using MD. A number of other simulations have focused instead on viral membrane association and fusion mechanisms (e.g., Rogers et al., 2015) and on general mechanisms of viral budding from cell membranes (Ruiz-Herrero and Hagan, 2015). There have been perhaps fewer simulation studies of viral envelope membranes per se. Here, we use coarse-grained (CG) MD simulations (Marrink et al., 2007) to incorporate lipids to the dengue envelope structure, and to explore the structural and biophysical properties of the complete model of the dengue envelope membrane. Our results provide insights into the viral protein-lipid interactions at the near-atomic level. The structure of dengue virion coated with therapeutic antibodies has recently been determined (Fibriansah et al., 2015). Thus, the establishment of a computational model offers future possibilities for the in silico exploration of the dynamic properties of viral envelopes for therapeutic insights.

## Results and Discussion

**Figure 1:**
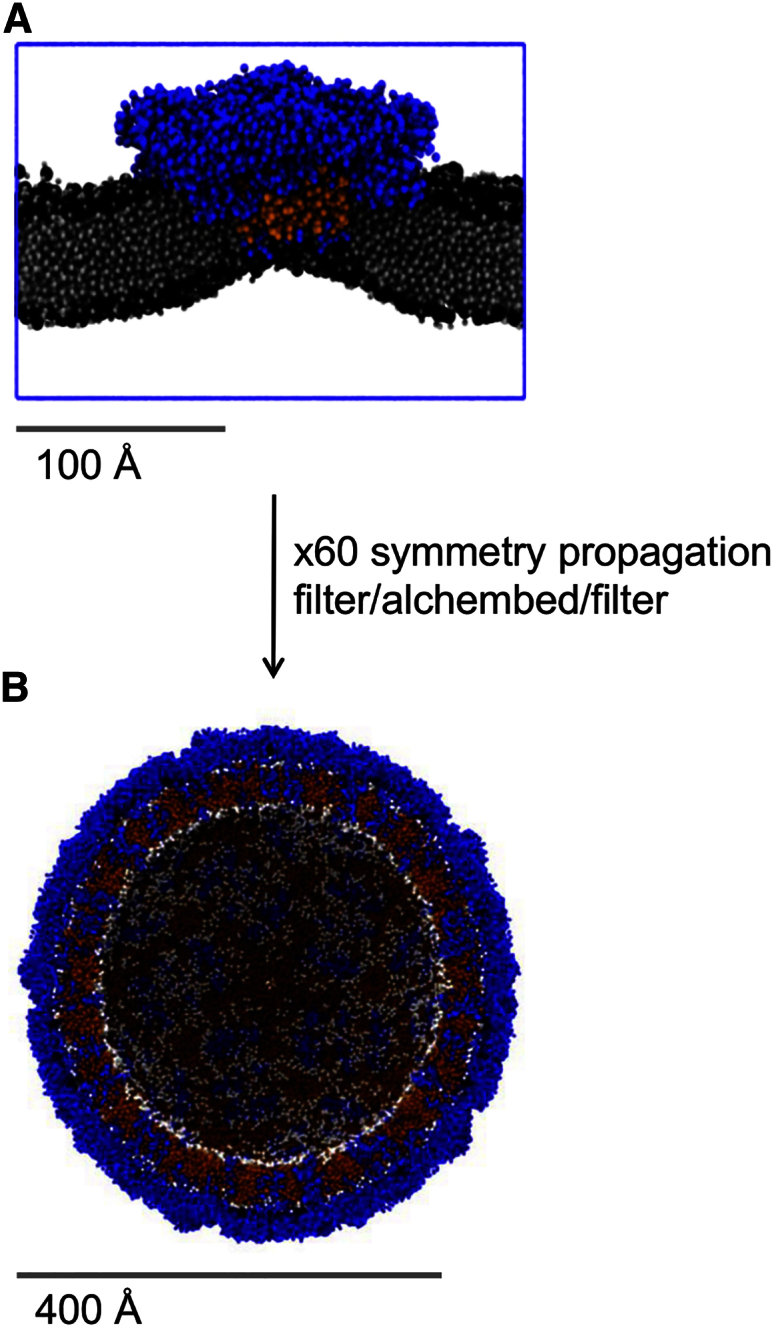
Modelling the Lipid Bilayer of the Dengue Virion Envelope (A) Dengue asymmetric unit equilibrated in a PC bilayer (TM domains in orange; remainder of protein blue; lipid in black/gray). (B) The virion envelope is cut in half, revealing both the protein (blue) and lipid (white and orange) components.

Our computational model of the outer envelope of the dengue virion (Figure 1) includes seven lipid species based on the lipidome of virus-infected mosquito cells (Perera et al., 2012), namely: palmitoyloleoylphosphatidylcholine (POPC), dilinoleylphosphatidylcholine (DUPC), dipalmitoylphosphatidylethanolamine (DPPE), dioleoylphosphatidylserine (DOPS), palmitoylsphingomyelin with a choline headgroup (PPCS), palmitoylsphingomyelin with an ethanolamine headgroup (PPCE), and ceramide with two C16 tails (CER), amounting to ∼8,000 lipid molecules in total (see Supplemental Information for details). Note that cholesterol was *not* present, as it was not quantified in the above lipidomics study. This model was generated starting from a single asymmetric unit containing three E proteins and three M proteins of the dengue protein envelope which was simulated in a planar bilayer containing a single lipid species (phosphatidylcholine [PC]; Figure 1A). This initial 0.5-μs CG MD simulation revealed some local curvature of the bilayer, and also a degree of bilayer thinning adjacent to the transmembrane (TM) domains of the E and M proteins (discussed in more detail below). The resultant model of the E_3_M_3_ asymmetric unit in a PC bilayer was then propagated using the viral icosahedral symmetry operations to yield an initial model of the virion with a purely PC lipid bilayer (Figure 1B). The lipids of the model were then edited to match the insect cell lipidome (see Experimental Procedures for details). This model was then used as the basis of an extended (5 μs) CG MD simulation of the dengue virion. This simulation used the MARTINI CG force field (Monticelli et al., 2008), which has been widely applied for membranes and membrane proteins (Marrink and Tieleman, 2013) and which successfully reproduces experimental parameters for large viral and lipid vesicle systems (Louhivuori et al., 2010, Reddy et al., 2015).

**Figure 2:**
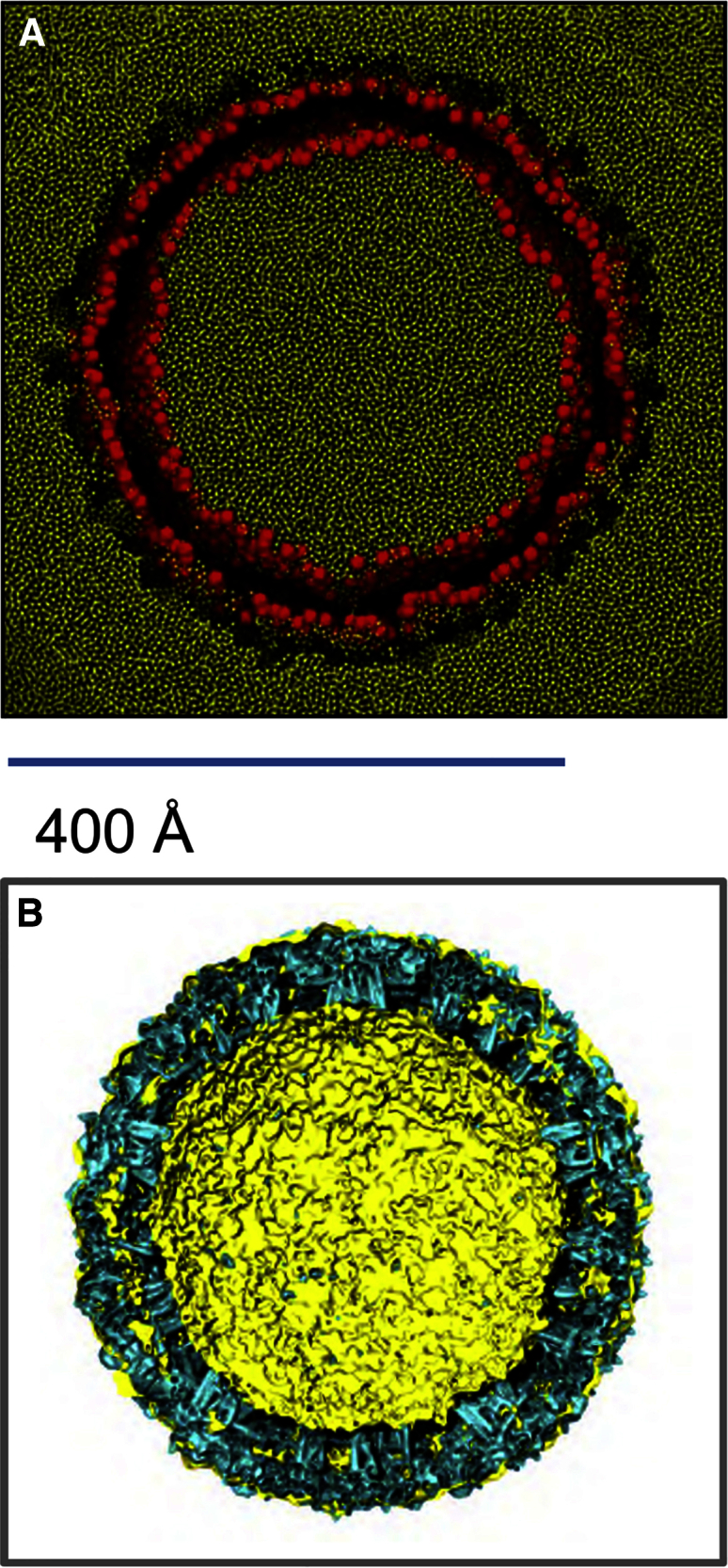
Simulation of the Dengue Virion Envelope (A) Water particles shown in a cross section through the dengue virion simulation (showing the final frame coordinates). The outer protein shell has been excluded for clarity, water is shown in yellow, and lipid headgroup particles are in orange. Note that in this simulation the nucleocapsid was replaced by CG water particles within the interior of the virion. (B) Superposition of the final snapshot of the dengue coarse-grain simulation (yellow; surface representation) and the isosurface of the whole dengue virion obtained from cryo-EM (cyan; EMD-5520; http://www.ebi.ac.uk/pdbe/entry/emdb/EMD-5520). Note that the simulation model did not include the surface glycans observed by cryo-EM.

The virion envelope model excluded the nucleocapsid, which was replaced by CG water particles within the interior of the virion. The nucleocapsid is not well defined in structures of the dengue virion. It has a lower degree of order and a smaller radius than in, e.g., alphaviruses, and no specific interaction between nucleocapsid and the outer glycoprotein scaffold has been demonstrated for flaviviruses (Kuhn et al., 2002). The simulated envelope model exhibited a stable outer diameter (480 ± 2 Å compared with ∼500 Å by cryo-EM, at 24 Å resolution [Kuhn et al., 2002]), shape (i.e. sphericity; Figure S1A), and lipid-protein interactions (Figure S1B) over the course of the 5-μs simulation. This is comparable with the behavior of, e.g., an influenza A virion model in microsecond-duration CG simulations (Reddy et al., 2015). At the end of the dengue virion simulation, a number of water particles were observed to remain between the outer protein shell and the lipid bilayer underneath (possibly forming bridging interactions). However, water was largely excluded from the hydrophobic core of the bilayer (Figure 2A), which therefore was judged to have maintained its integrity over the course of the simulation. A simple superposition of the final snapshot of the dengue CG simulation and of the isosurface of the whole dengue virion obtained from cryo-EM (EMD-5520; Figure 2B) shows (as might be anticipated) a good overall match between the size and shape of the experimentally determined density and the simulated structure.

**Figure 3:**
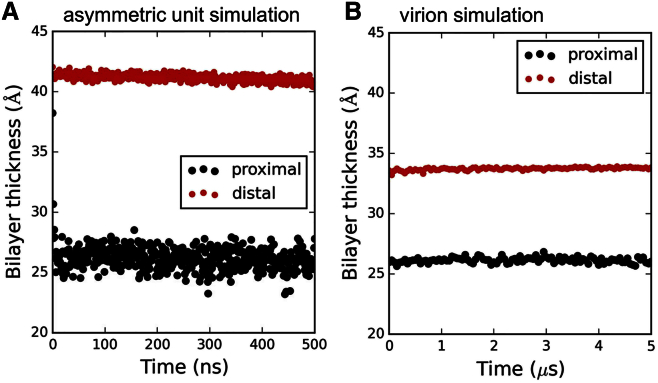
Thickness of the Lipid Bilayer (A) Bilayer thickness proximal (i.e., within 6 Å) and distal to the protein during a short (0.5 μs) simulation of the 3E + 3M asymmetric unit in a PC bilayer. (B) Bilayer thickness proximal and distal to protein TM domains over the course of the 5-μs duration full dengue virion simulation. See also Figures S1 and S2.

Bilayer thinning local to the TM domains of the proteins was seen in the full virion simulations. The degree of thinning (assessed as the difference between the thickness of the bilayer distal from and proximal to the protein) was 7.6 ± 0.3 Å. This is somewhat lower than the corresponding degree of thinning that was observed in the simulations of a single E_3_M_3_ asymmetric unit in a PC bilayer (see above), reflecting a general reduction of ∼5 Å in overall bilayer thickness in the virion envelope simulations (Figure 3). However, it remains clear that in both the E_3_M_3_ asymmetric unit + lipid simulation and also in the full envelope simulations, the bilayer thickness local to the protein was ∼25 Å. This agrees well with previous cryo-EM estimates of the protein-local bilayer thickness of between 25 and 30 Å in dengue virions (Laurinmaki et al., 2005, Zhang et al., 2013a).

**Figure 4:**
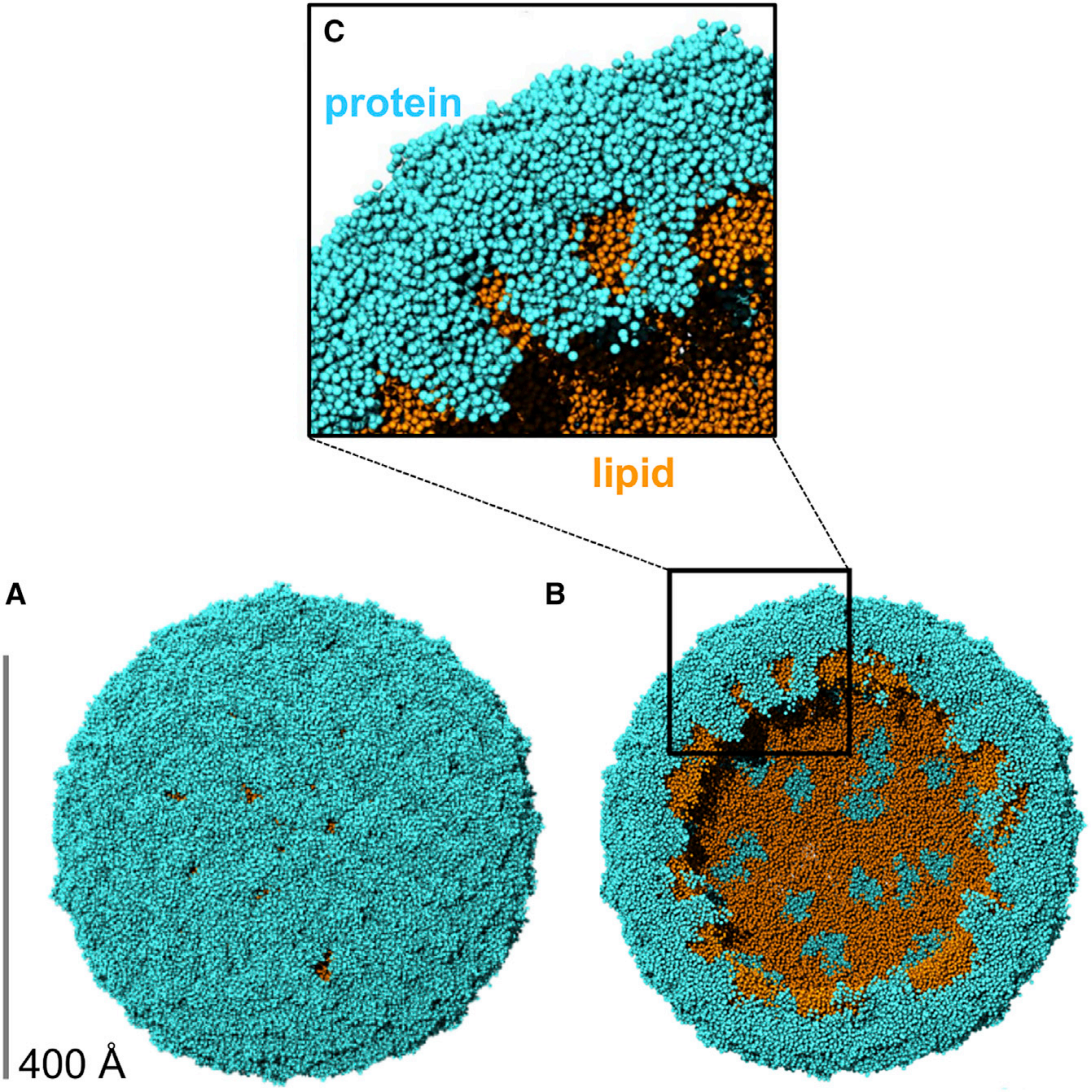
Proteins and Lipids in the Envelope The virion envelope (A) is cut in half (B) to reveal both the protein (cyan) and lipid (orange) components. A zoomed-in view (C) reveals close and extensive interactions of protein and lipids.

The membrane proteins of the dengue virion model contribute a total of 720 TM helices, which form extensive interactions with the bilayer lipids (Figure 4). The TM helices correspond to ∼18% of the membrane surface area in each leaflet (Figure S2). This approaches the degree of crowding observed in mammalian cell membranes, with, e.g., more than 20% of the cross-sectional area of red blood cell membranes and synaptic vesicle membranes being TM proteins (Dupuy and Engelman, 2008). Thus the dengue virus envelope membrane is more crowded (with protein) than, e.g., that of influenza A, where the TM domains occupy ∼2% of the surface area of the virion envelope (Figure S2). Furthermore, the outer surface of the dengue lipid bilayer is almost completely covered by the surrounding shell of (E + M) proteins (Figure 4A). This provides an unusual biophysical environment for the underlying membrane because of the large contact area between protein and lipid, resulting in the potential for attenuation of lipid diffusion via extensive protein-lipid interactions. Previous MD simulation studies of enveloped virions have focused on viruses that have substantially more lipids exposed to the surrounding solvent (Ayton and Voth, 2010, Reddy et al., 2015). For example, HIV-1 only displays 14 ± 7 glycoprotein spikes per virion (Zhu et al., 2006), and influenza A glycoproteins are separated by large (∼125 Å) solvent-exposed lipid areas on the envelope in both experimental and computational studies (Reddy et al., 2015, Wasilewski et al., 2012). Dengue virus thus differs from both HIV-1 and influenza A (and from host cell membranes) in that in dengue 68% of the outer leaflet lipid headgroups are in contact with a surrounding envelope protein (Figure S1B).

**Figure 5:**
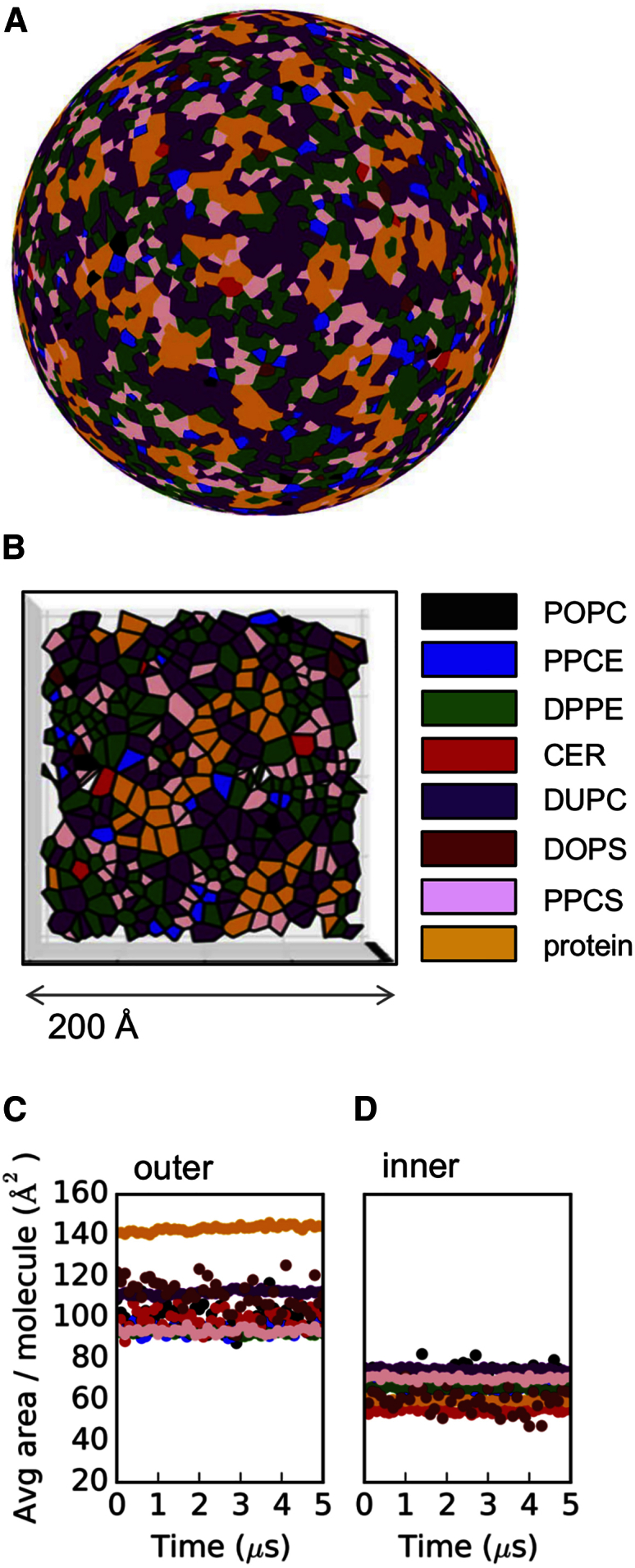
Lipid Surface Areas Dengue virion outer leaflet spherical Voronoi diagram (A and B) and average area per molecule tracked for outer (C) and inner (D) leaflets. Molecules are colored as follows: POPC (palmitoyloleoylphosphatidylcholine; black), PPCE (palmitoylsphingomyelin with an ethanolamine headgroup; blue), DPPE (dipalmitoylphosphatidylethanolamine; green), CER (ceramide with two C16 tails; red), DUPC (dilinoleylphosphatidylcholine; purple), DOPS (dioleoylphosphatidylserine; brown), PPCS (palmitoylsphingomyelin with a choline headgroup; pink), and protein (yellow).

The lateral distribution of lipids in the two leaflets of the dengue envelope was unexpected. The average area per molecule values were calculated using spherical Voronoi diagrams and tracked over the course of the 5-μs simulation for each lipid species (Figure 5). The outer leaflet mean area per lipid is ∼90–110 Å^2^, whereas the inner leaflet values range from ∼50 to 80 Å^2^. The dengue lipids are thus less densely packed than in influenza A, for which outer and inner leaflet area per lipid values ranged between ∼40 and 60 Å^2^ and ∼40 and 50 Å^2^, respectively (although the low values for the influenza virus membrane may in part also reflect the high cholesterol content of the bilayer; Leftin et al., 2014). Dengue lipids in the outer leaflet are also less densely packed than lipids in previously reported CG simulations of 1,2-dimyristoyl-*sn*-glycero-3-phosphocholine (DMPC) vesicles, where the area per DMPC molecule was ∼63 Å^2^ (Braun and Sachs, 2014).

This idiosyncratic lateral organization of the outer leaflet lipids correlates with their extensive interactions with the external shell of envelope proteins covering the surface of the dengue virion. Indeed, ∼68% of outer leaflet lipid headgroups are in contact with protein, compared with ∼22% of inner leaflet headgroups (Figure S1B). The marked bilayer distortions and thinning (described above) in dengue envelope areas that are proximal to the short TM domains may also contribute to the differential lipid density when compared with more conventionally organized viral envelopes and cell membranes.

**Figure 6:**
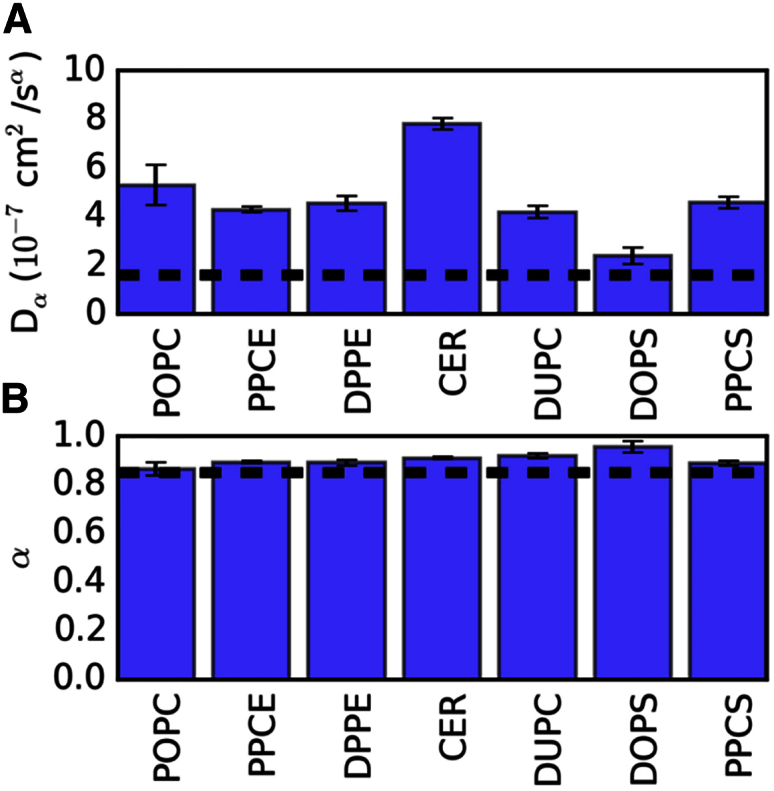
Lipid Diffusion Diffusion constants (A) and scaling exponents (B) for the lateral movement of lipids in the dengue virion. Dashed lines represent averages from influenza A. Error bars are standard deviations. Abbreviations as in Figure 5.

**Figure 7:**
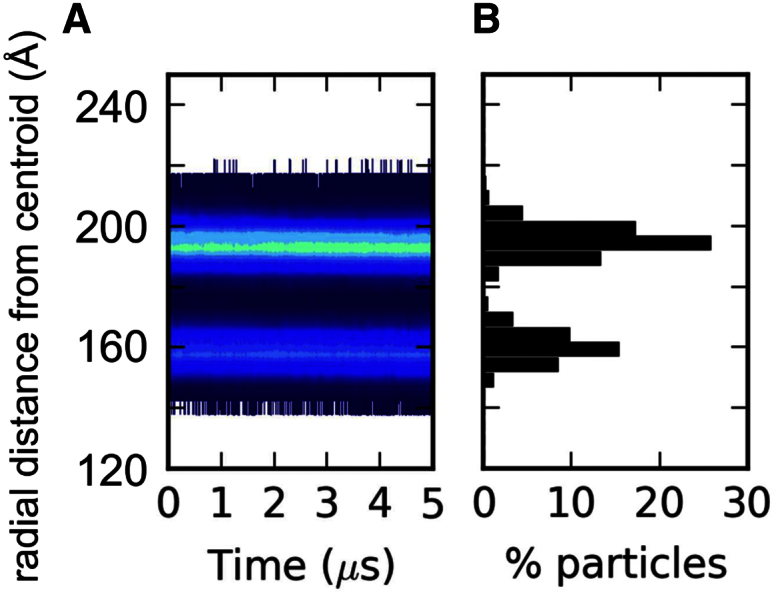
Distribution of Sphingomyelin in the Dengue Virion Envelope (A) The headgroup PO4 particle radial distances from the virion centroid are displayed with the color contour plot on a logarithmic scale. (B) The radial distance histogram at the end of the simulation.

Given the static (time-averaged) organization of dengue envelope lipids, their dynamic properties such as diffusional mobility are also of substantial interest for comparison with other viruses. Lateral diffusion of all seven lipid species in the dengue model was anomalous, with an overall scaling exponent (α) of ∼0.9 (Figure 6; Equation 1). The anomalous diffusion coefficients (*D*_α_) ranged from ∼2 to 8 × 10^−7^ cm^2^/s^α^. Interestingly, similar α and *D*_α_ values were previously reported for influenza A simulations (Reddy et al., 2015) and experiments (Polozov et al., 2008). Thus, despite the absence of cholesterol from the dengue lipid bilayer, dengue exhibits lipid mobility similar to that of the “raft-like” bilayer (which contains >50% cholesterol) of the influenza A envelope. This suggests that the density of TM domains within the bilayer plus near-complete coverage of the outer leaflet of the lipid bilayer by the outer protein shell in the dengue virion may confer a degree of biophysical robustness (i.e., slow lipid diffusion alongside stable bilayer thickness, virion diameter, and shape). In contrast, in influenza viruses the robustness is provided by the high cholesterol content of the envelope lipids (Reddy et al., 2015). Furthermore, the distribution of sphingolipid along the bilayer normal in the dengue virion was quite broad (Figure 7). This is consistent with the broadened sphingolipid distribution observed for influenza A when its proteins were restrained to mimic the M1 matrix layer underneath the envelope. Thus, the outer protein shell of the dengue virion, where proteins are interlocked in an icosahedral arrangement, appears to mimic the proposed immobilizing effect of the inner M1 matrix protein shell of influenza A (Veit and Thaa, 2011).

Many enveloped viruses (i.e., HIV [Brugger et al., 2006] and influenza [Gerl et al., 2012, Ivanova et al., 2015]) select a specific lipid composition distinct from the overall membrane lipid composition of the host cell. In the current study, the dengue lipidome was modeled based on that of an insect host cell membrane. It is possible that changes in the details of our dengue envelope lipidome model, should the lipidome of the virion membrane become available, might lead to some changes in the area per molecule and other parameters, especially as dengue budding occurs into the lumen of the ER, at least in human cells (den Boon and Ahlquist, 2010). However, it seems unlikely that the overall structural and biophysical properties observed in the current studies would be substantially different.

Our simulations are of the “smooth” mature form of the virus. It will be of interest to compare this biophysical behavior of dengue with that of the “bumpy” outer protein shell configuration(s) (Fibriansah et al., 2015, Zhang et al., 2013b) where there is a greater exposure of the lipid bilayer to bulk water. In this way, one may ultimately be able to relate changes in biophysical properties of the viral envelope lipid bilayer to the different clinical isolates and stages of the life cycle of dengue virus. It should also be possible to enhance our model to include recently resolved structures of N-glycans (Lei et al., 2015) and also to explore possible models of dengue virus binding to target cell surfaces (Cruz-Oliveira et al., 2015) by combining our viral model with recent plasma membrane models (Ingolfsson et al., 2014, Koldsø et al., 2014) in large-scale simulations.

### Conclusions

We have constructed a computational model of the dengue virion by combining the known structure of the membrane proteins with a CG model of the lipid bilayer, the composition of which was derived from lipidomics data for insect cells. This integrated computational model is robust in extended (microsecond) MD simulations and maintains a bilayer with slow anomalous lateral diffusion of lipids, which could be evaluated by, e.g., solid-state nuclear magnetic resonance, as has been done for influenza virion membranes (Polozov et al., 2008). Thus, the outer protein coat of the dengue virion confers biophysical robustness to the envelope by means of physical interaction with the lipids (∼70% dengue outer leaflet headgroups within 6 Å of protein), in contrast to the influenza A envelope where robustness is provided directly in its raft-like lipidome.

## Experimental Procedures

### Construction of Dengue Virion Protein-Lipid Envelope

A 3.5-Å resolution atomic structure of the asymmetric unit of the insect-grown dengue virus type 2 (Thailand/PUO-218/1980) outer protein envelope (PDB: 3J27; Zhang et al., 2013a) was prepared for conversion to a CG representation by removal of surface glycans and any terminal oxygen atoms, which would otherwise complicate coarse graining. Coarse graining was performed to match the MARTINI 2.1 force field particle mappings (Marrink et al., 2007). Steepest descent energy minimization was performed using GROMACS 4.5 (Hess et al., 2008) (www.gromacs.org) and the minimized CG coordinates of the asymmetric unit included 3,684 particles (3 × 158 M protein particles + 3 × 1,070 E protein particles). The third eigenvector of all CG particles in the asymmetric unit was aligned along the +z axis of the coordinate system to orient the 12 TM domains of the asymmetric unit along the same axis in preparation for lipidation in the xy plane. A POPC CG bilayer (1,717 molecules) was self-assembled at 323 K as previously described (Reddy and Rainey, 2012) and used as a template for embedding the asymmetric unit at various bilayer burial depths using the *g_membed* tool (Wolf et al., 2010). Specifically, the centroid of the asymmetric unit TM domains was placed between 0 and 15 Å (inclusive) above the phosphate centroid of the bilayer at 3-Å intervals, followed by the *g_membed* procedure and 500 ns of equilibration in a hydrated and neutralized system at each burial depth (10-fs time steps, 323 K, approximately 47,000 water (W) particles, three Cl^−^ ions; final box dimensions approximately 240 × 240 × 140 Å^3^). The most suitable equilibrated configuration (9 Å starting elevation) was selected based on similarity (by visual inspection) of the lipid bending around the asymmetric unit to the lipid electron density map around the E:M:M:E heterotetramer previously reported (Zhang et al., 2013a).

The equilibrated CG asymmetric unit in a POPC bilayer was translated and rotated such that its protein coordinates matched the CG coordinates of the first asymmetric unit in the biological assembly of the original structure with minimal root-mean-square deviation (RMSD). The repositioned coordinates (protein and lipid) represent the first of 60 asymmetric units, and the remaining 59 units (and their associated lipids) were propagated using the icosahedral symmetry translation/rotation operations specified in the biological assembly instructions in the original structure. The symmetry operations produce substantial steric conflicts because the lipid bilayer is larger than the asymmetric unit contained within it, leading to lipid-lipid and lipid-protein spatial overlap of adjacent asymmetric units. A combination of our in-house *Alchembed* procedure (Jefferys et al., 2015) and selective trimming of overhang lipids was iteratively applied until all intermolecular steric conflicts (2 Å cutoff) were resolved.

The POPC CG dengue construct (dengue E/M protein shell, 8,224 POPC molecules, 602,410 W particles, 180 Cl^−^ particles, 31,715 WF [antifreeze water] particles) was equilibrated (10-fs time step, 323 K) with GROMACS 4.5.x (104 ns) or 4.6.x (273 ns). Lipid and protein RMSD relative to the starting configuration stabilized as did their respective sphericity values, and similar behavior was observed for both versions of GROMACS despite their differing electrostatics algorithms.

POPC PO4 headgroup particles were then categorized into leaflets using a 180-Å radial distance threshold in the absence of protein, and vectors were defined from the virion centroid to the outer leaflet PO4 particles and in the opposite direction for inner leaflet PO4 particles. Lipid molecule templates from the host lipidome (Perera et al., 2012) were then aligned such that the vector connecting their centroid to a headgroup particle was parallel to a randomly selected POPC alignment vector in a given leaflet, and the POPC molecule was replaced by the transformed template lipid. Proteins were then reincorporated into the outer envelope model, and steric conflicts between molecules were resolved by an alchemical particle regrowth procedure and the removal of 349 lipids involved in contacts within a 2.0-Å cutoff. The final coordinates were free of steric conflicts, and the model analyzed here (1.03 M particles total) consists of 180 E proteins, 180 M proteins, 77 POPC, 319 PPCE, 2,412 DPPE, 420 CER, 3,117 DUPC, 63 DOPS, 1,467 PPCS, 682,910 W (water), 117 Cl^−^, and 35,948 WF particles.

### Lipid-Protein Contact Analysis

In each parsed frame of the simulation trajectory, lipid headgroup coordinates and all protein particle coordinates were adjusted such that the centroid of the lipid headgroups was translated to the origin. The lipid headgroup Cartesian coordinates were converted to spherical polar coordinates and sorted by radial distance after accounting for any residual lipids outside of the virion envelope and surrounded by bulk solvent. The average of the lipid headgroup radial distance minimum and maximum was used as the threshold for leaflet assignment (with confirmation of leaflet assignments by visual inspection at 1-μs trajectory intervals). Separate distance matrices were calculated between all lipid headgroup particles in a given leaflet and the full set of protein particles in the system. The percentage of lipid headgroup particles in each leaflet that were within 6 Å of at least one protein particle was reported.

### Assessment of Virion Stability

In each parsed frame of the simulation trajectory, the full set of dengue protein coordinates was translated to place the centroid at the origin. The protein Cartesian coordinates were converted to spherical polar coordinates, and the average value of the 60 largest radial distances was calculated. The outer diameter was estimated as double the latter average distance. The shape of the virion was assessed using the sphericity parameter (Wadell, 1935) and an algorithm previously described (Reddy et al., 2015).

### Lipid Stratification Tracking Analysis

We employed an algorithm similar to that previously described for this type of analysis (Reddy et al., 2015). In brief, for each parsed simulation frame the centroid of the protein and lipid species was calculated. A distance matrix was calculated between the system centroid and the lipid headgroups and histogrammed in 5-Å bins between 0 and 800 Å. The results are reported as radial distance contour plots on a logarithmic scale for each lipid species.

### Lipid Diffusion Analysis

We employed an algorithm similar to that previously described using our documented open-source code for analyzing diffusion (http://dx.doi.org/10.5281/zenodo.11827). Individual lipid centroid mean-square displacement values were calculated over the range of window sizes including: 1, 3, 5, 10, 25, 50, 100, 200, 300, 400, and 500 ns. Diffusion constants and scaling exponents were estimated using non-linear least-squares fitting to the two-parameter equation described previously (Kneller et al., 2011):

(Equation 1) MSD = 4*D*_α_*t*^α^,

where 0 < α < 2.

The SD of both parameters was obtained from the square root of the diagonal of the covariance matrix from the non-linear least-squares fit.

### Area per Molecule Analysis

The lipids in the dengue virion were assigned to leaflets using a midpoint distance threshold, and projected onto spheres representing the average radii of the inner and outer leaflets of the virion. The centroids of individual protein TM domains were projected both upward and downward to approximate the footprint of the protein in each leaflet. The Delaunay triangulation of the generators (original data points) representing the lipid and protein species in each leaflet was obtained by calculating their convex hull (Caroli et al., 2010). Spherical Voronoi diagrams were generated and used to accurately parse the area per molecule on the surface of the virion. Documented open-source code is available (http://dx.doi.org/10.5281/zenodo.13688), and this method will be described in detail in another paper (T.R. and M.S.P.S., unpublished data).

### Bilayer Thickness Analysis

For each parsed frame of the asymmetric unit equilibration simulation in a POPC bilayer, a distance matrix was calculated between phosphate headgroup particles in each leaflet (assigned based on z coordinates in first frame) and all protein coordinates. The lipid headgroups in each leaflet were then categorized as protein-local or -distal using a 6-Å distance threshold. The average z coordinates of all four groups were calculated, and the differences in the protein-local and protein-distal z coordinates of each leaflet were used to estimate bilayer thickness values. A similar approach was used for the dengue outer envelope simulation, except that distance matrices for assessment of protein proximity employed only the TM domain particles of the proteins (to avoid capturing most of the outer leaflet as “protein-local”). Leaflet assignments for lipid headgroups and the thickness values were calculated using radial distances from spherical polar coordinates.

### Simulation Details

The dengue virion model was equilibrated for 5 μs at 323 K using the MARTINI 2.1 force field (Marrink et al., 2007) and GROMACS 4.6 (Hess et al., 2008), after which another 5-μs simulation (used for analyses) was performed using the same parameters. Equilibration was deduced on the basis of stable virion outer diameter and shape (sphericity). The simulations were performed using 10-fs time steps, frames written at 0.1-ns intervals, electrostatics treated as reaction field with Coulomb cutoff of 11 Å using a potential-shift modifier and the Verlet cutoff scheme, and Lennard-Jones cutoff of 11 Å. Protein, lipid, and solvent were separately temperature coupled using the Berendsen algorithm (1.0 ps time constant) (Berendsen et al., 1984), and isotropic pressure coupling was employed using the Berendsen algorithm with a time constant of 1.1 ps and compressibility of 1 × 10^−6^ bar^−1^.

### Analysis and Visualization Tools

Simulation trajectories were exposed using the Python MDAnalysis library (Michaud-Agrawal et al., 2011) and parsed with open-source Python libraries including numpy (van der Walt et al., 2011), scipy (Oliphant, 2007), pandas (McKinney, 2010), matplotlib (Hunter, 2007), and IPython (Perez and Granger, 2007). Visualizations were performed using VMD (Humphrey et al., 1996) and PyMOL (www.pymol.org).

## Author Contributions

T.R. designed and performed the simulations and data analysis. T.R. drafted the manuscript, and M.S.P.S. commented on and helped to revise it. M.S.P.S. supervised the studies.

## Supplemental Information

Document S1. Supplemental Computational Procedures and Figures S1 and S2

dengue_final_snapshot_no_solvent.pdb

Document S2. Article plus Supplemental Information
