# Supplementary material for: The Role of the Membrane in the Structure and Biophysical Robustness of the Dengue Virion Envelope
Source: Structure. 2016 Mar 1;24(3):375–82. doi: 10.1016/j.str.2015.12.011 (PMC4780862; doi:10.1016/j.str.2015.12.011)
Supplement: Document S1. Supplemental Computational Procedures and Figures S1 and S2 [file mmc1.pdf]

Structure, Volume *24*

Supplemental Information

The Role of the Membrane in the Structure and Biophysical Robustness of the Dengue Virion Envelope

Tyler Reddy and Mark S.P. Sansom

Supporting Information for:

The Role of the Membrane in the Structure and Biophysical Robustness of the Dengue Virion Envelope

*Tyler Reddy & Mark S P Sansom**

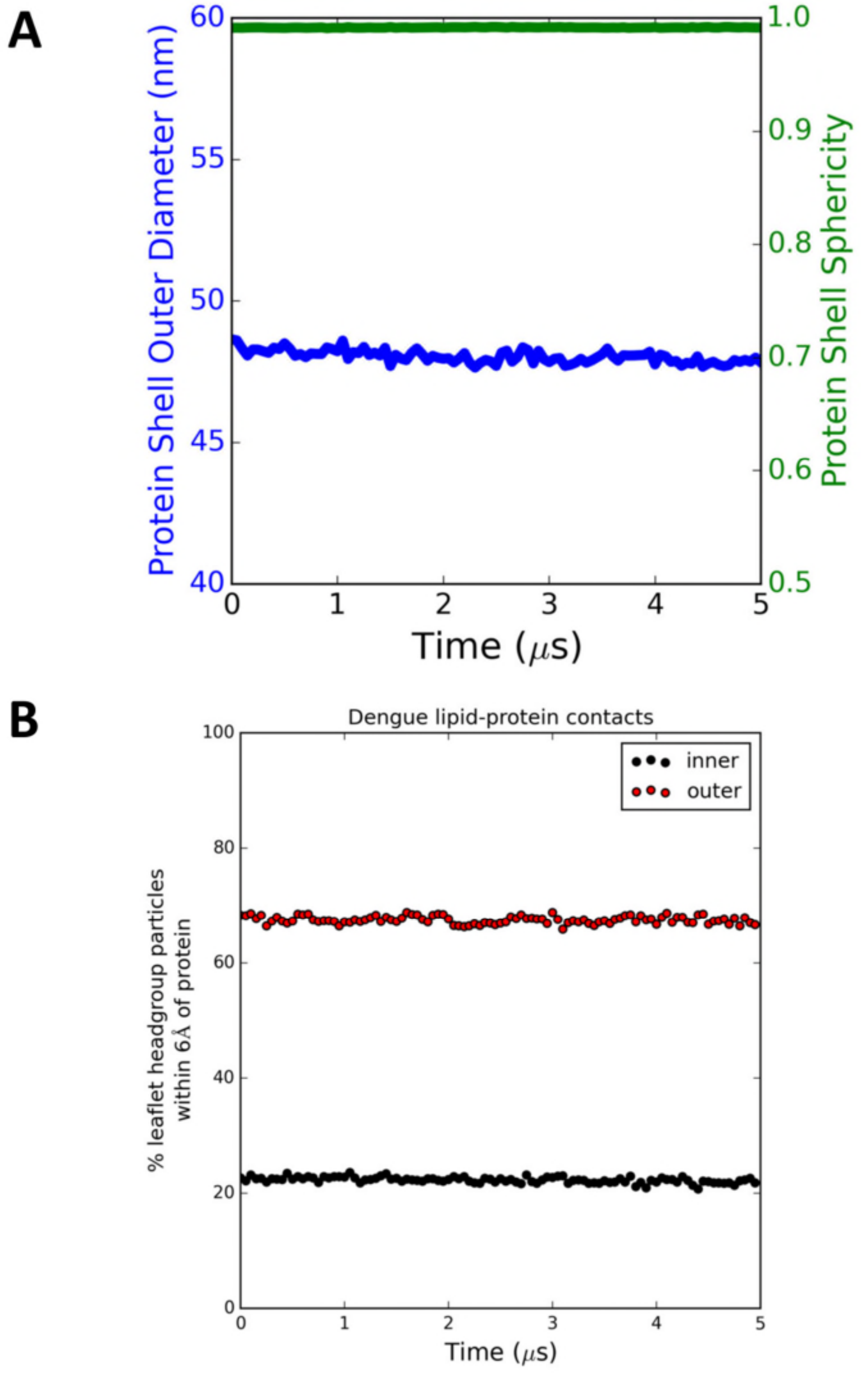

Figure S1, related to Figures 1 and 2: (A) Outer diameter and shape (i.e. sphericity); and (B) lipid-protein interactions (calculated as the fraction of lipid headgroup particles in contact with protein) over the course of a 5 μs CG MD simulation of the dengue virion model. As discussed above, the the sphericity Ψ is defined as the ratio of the surface area of a sphere with the same volume as the particle (V_P) to the surface area of the particle (A_P).

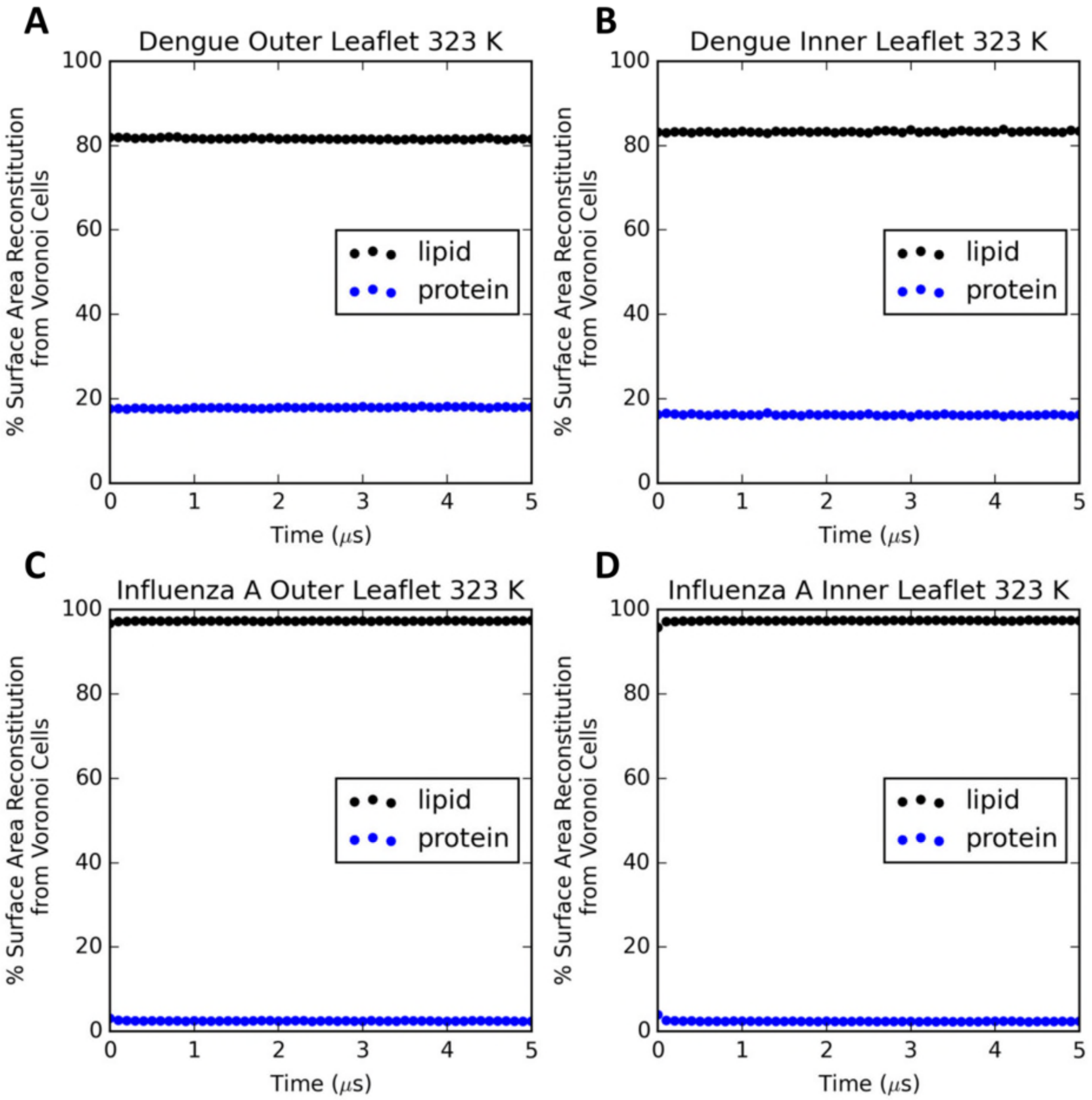

Figure S2, related to Figure 4: Assessment of relative surface area occupied by proteins (blue) and lipids (black) for the dengue virion (A, B) and influenza A virion (C, D) calculated based on the sum of the Voronoi cell areas. The dengue virion surface is normally covered by protein, and the values reported here assume a peel-away view that retains only the TMDs of the surface proteins. The influenza A simulation was previously reported (Reddy et al., 2015), but was not analyzed in this manner.

Supplemental Computational Procedures

Construction of dengue virion protein-lipid envelope

A 3.5 Å resolution atomic structure of the asymmetric unit of the insect-grown dengue virus type 2 (Thailand/PUO-218/1980) outer protein envelope [PDB: 3J27] (Zhang et al., 2013) was prepared for conversion to a coarse-grained representation by removal of surface glycans and any terminal oxygen atoms (OXT), which would otherwise complicate coarse-graining. Coarse-graining was performed to match the MARTINI 2.1 forcefield particle mappings (Marrink et al., 2007). Steepest descent energy minimization was performed using GROMACS 4.5 (Hess et al., 2008) (www.gromacs.org) and the minimized CG coordinates of the asymmetric unit included 3684 particles (3 x 158 M protein particles + 3 x 1070 E protein particles). The third eigenvector of all CG particles in the asymmetric unit was aligned along the $+z$ axis of the coordinate system in order to orient the 12 TMDs of the asymmetric unit along the same axis in preparation for lipidation in the xy plane. A POPC CG bilayer (1717 molecules) was self-assembled at 323 K as previously described (Reddy and Rainey, 2012) and used as a template for embedding the asymmetric unit at various bilayer burial depths using the *g_membed* tool (Wolf et al., 2010). Specifically, the centroid of the asymmetric unit TMDs was placed between 0 and 15 Å (inclusive) above the phosphate centroid of the bilayer at 3 Å intervals, followed by the *g_membed* procedure and 500 ns of equilibration in a hydrated and neutralized system at each burial depth (10 fs timesteps, 323K, approx. 47000 W molecules, 3 Cl^- ions; final box dimensions approx. 240 x 240 x 140 $Å^3$). The most suitable equilibrated configuration (9 Å starting elevation) was selected based on similarity (by visual inspection) of the lipid bending around the asymmetric unit to the lipid electron density map around the E:M:M:E heterotetramer previously reported (Zhang et al., 2013).

The equilibrated CG asymmetric unit in a POPC bilayer was translated and rotated such that its protein coordinates matched the CG coordinates of the first asymmetric unit in the biological assembly of the original structure with minimal RMSD. The repositioned coordinates (protein and lipid) represent the first of 60 asymmetric units, and the remaining 59 units (and their associated lipids) were propagated using the icosahedral symmetry translation / rotation operations specified in the biological assembly instructions in the original structure. The symmetry operations produce substantial steric conflicts because the lipid bilayer is larger than the asymmetric unit contained within it, leading to lipid-lipid and lipid-protein spatial overlap of adjacent asymmetric units. A combination of our in-house *Alchembed* procedure (Jefferys et al., 2015) and selective trimming of overhang lipids was iteratively applied until all intermolecular steric conflicts (2 Å cutoff) were resolved.

The POPC CG dengue construct (dengue E/M-protein shell, 8224 POPC molecules, 602410 W molecules, 180 Cl^- particles, 31715 WF particles) was equilibrated (10 fs timestep, 323 K) with GROMACS 4.5.x (104 ns) or 4.6.x (273 ns). Lipid and protein RMSD relative to the starting configuration stabilized as did their respective sphericity values, and similar behaviour was observed for both versions of GROMACS despite their differing electrostatics algorithms.

POPC PO4 headgroup particles were then categorized into leaflets using a 180 Å radial distance threshold in the absence of protein, and vectors were defined from the virion centroid to the outer leaflet PO4 particles and in the opposite direction for inner leaflet PO4 particles. Lipid molecule templates from the host lipidome (Perera et al., 2012) were then aligned such that the vector

connecting their centroid to a headgroup particle was parallel to a randomly-selected POPC alignment vector in a given leaflet, and the POPC molecule was replaced by the transformed template lipid. Proteins were then reincorporated into the outer envelope model and steric conflicts between molecules were resolved by an alchemical particle regrowth procedure and the removal of 349 lipids involved in contacts within a 2.0 Å cutoff. The final coordinates were free of steric conflicts and the model analysed here (1.03 M particles total) consists of 180 E proteins, 180 M proteins, 77 POPC (palmitoyl oleoyl phosphatidylcholine), 319 PPCE (palmitoyl sphingomyelin with ethanolamine headgroup), 2412 DPPE (dipalmitoyl phosphatidylethanolamine), 420 CER (ceramide with two C16 tails), 3117 DUPC (dilinoleyl phosphatidylcholine), 63 DOPS (di-oleoyl phosphatidylserine), 1467 PPCS (palmitoyl sphingomyelin with choline headgroup), 682910 W (water), 117 Cl-, and 35948 WF (antifreeze water) molecules.

Lipid-protein contact analysis

In each parsed frame of the simulation trajectory lipid headgroup coordinates and all protein particle coordinates were adjusted such that the centroid of the lipid headgroups was translated to the origin. The lipid headgroup Cartesian coordinates were converted to spherical polar coordinates and sorted by radial distance after accounting for any residual lipids outside of the virion envelope and surrounded by bulk solvent. The average of the lipid headgroup radial distance minimum and maximum was used as the threshold for leaflet assignment (with confirmation of leaflet assignments by visual inspection at 1 microsecond trajectory intervals). Separate distance matrices were calculated between all lipid headgroup particles in a given leaflet and the full set of protein particles in the system. The percentage of lipid headgroup particles in each leaflet that were within 6 Å of at least one protein particle was reported.

Assessment of virion stability

In each parsed frame of the simulation trajectory the full set of dengue protein coordinates was translated to place the centroid at the origin. The protein Cartesian coordinates were converted to spherical polar coordinates and the average value of the 60 largest radial distances was calculated. The outer diameter was estimated as double the latter average distance. The shape of the virion was assessed using the sphericity parameter (Wadell, 1935), and an algorithm previously described (Reddy et al., 2015). Thus the sphericity Ψ is defined as the ratio of the surface area of a sphere with the same volume as the particle (V_P) to the surface area of the particle (A_P):

$$\Psi = \frac{\pi^{1/3}(6V_P)^{2/3}}{A_P}.$$

Lipid stratification-tracking analysis

We employed an algorithm similar to that previously described for this type of analysis (Reddy et al., 2015). Briefly, for each parsed simulation frame the centroid of the protein and lipid species was calculated. A distance matrix was calculated between the system centroid and the lipid headgroups and histogrammed in 0.5 nm bins between 0 and 80 nm. The results are reported as radial distance contour plots on a logarithmic scale for each lipid species.

Lipid diffusion analysis

We employed an algorithm similar to that previously described using our documented open source code for analysing diffusion (http://dx.doi.org/10.5281/zenodo.11827). Individual lipid centroid mean square displacement values were calculated over the range of window sizes including: 1, 3, 5, 10, 25, 50, 100, 200, 300, 400, and 500 ns. Diffusion constants and scaling exponents were estimated using non-linear least squares fitting to the two parameter equation described previously (Kneller et al., 2001):

$$MSD = 4D_{\alpha}t^{\alpha} \qquad \text{where} : 0 < \alpha < 2$$

The standard deviation of both parameters was obtained from the square root of the diagonal of the covariance matrix from the non-linear least squares fit.

Area per molecule analysis

The lipids in the dengue virion were assigned to leaflets using a midpoint distance threshold and projected onto spheres representing the average radii of the inner and outer leaflets of the virion. The centroids of individual protein TMDs were projected both upward and downward to approximate the footprint of the protein in each leaflet. The Delaunay triangulation of the generators (original data points) representing the lipid and protein species in each leaflet was obtained by calculating their convex hull (Caroli et al., 2009). Spherical Voronoi diagrams were generated and used to accurately parse the area per molecule on the surface of the virion. Documented open source code for this (https://github.com/scipy/scipy/pull/5232) is currently under review for incorporation into the well-established scipy library (Oliphant, 2007), and this method will be described in detail in another paper (Reddy & Sansom, ms. in preparation).

Bilayer thickness analysis

For each parsed frame of the asymmetric unit equilibration simulation in a POPC bilayer a distance matrix was calculated between phosphate headgroup particles in each leaflet (assigned based on Z coordinates in first frame) and all protein coordinates. The lipid headgroups in each leaflet were then categorized as protein-local or –distal using a 6 Å distance threshold. The average Z coordinates of all four groups were calculated, and the differences in the protein-local and protein-distal Z coordinates of each leaflet were used to estimate bilayer thickness values. A similar approach was used for the dengue outer envelope simulation, except that distance matrices for assessment of protein proximity employed only the TMD particles of the proteins (to avoid capturing most of the outer leaflet as 'protein-local'). Leaflet assignments for lipid headgroups and the thickness values were calculated using radial distances from spherical polar coordinates.

Simulation Details

The dengue virion model was equilibrated for 5 microseconds at 323 K using the MARTINI 2.1 forcefield (Marrink et al., 2007) and GROMACS 4.6 (Hess et al., 2008), and then another 5 microsecond simulation (used for analyses) was performed using the same parameters. The simulations were performed using 10 fs timesteps, frames written at 0.1 ns intervals, electrostatics treated as reaction field with Coulomb cutoff of 1.1 nm using a potential-shift modifier and the Verlet cutoff scheme, and Lennard-Jones cutoff of 1.1 nm. Protein, lipid and solvent were separately temperature coupled using the Berendsen algorithm (1.0 ps time constant) (Berendsen et

al., 1984), and isotropic pressure coupling was employed using the Berendsen algorithm with a time constant of 1.1 ps and compressibility of $1x10^{-6}$ bar^{-1}.

Analysis and Visualization Tools

Simulation trajectories were exposed using the Python MDAnalysis library (Michaud-Agrawal et al., 2011) and parsed with open-source Python libraries including numpy (van der Walt et al., 2011), scipy (Oliphant, 2007), pandas (McKinney, 2010), matplotlib (Hunter, 2007), and IPython (Perez and Granger, 2007). Visualizations were performed using VMD (Humphrey et al., 1996) and PyMOL (Schrodinger, 2010).

References

Berendsen, H.J.C., Postma, J.P.M., van Gunsteren, W.F., DiNola, A., and Haak, J.R. (1984). Molecular dynamics with coupling to an external bath. J. Chem. Phys. *81*, 3684-3690.

Caroli, M., M. M. de Castro, P., Loriot , S., Teillaud, M., and Wormser, C. (2009). Robust and Efficient Delaunay triangulations of points on or close to a sphere. [Research Report] RR-7004.

Hess, B., Kutzner, C., van der Spoel, D., and Lindahl, E. (2008). GROMACS 4: algorithms for highly efficient, load-balanced, and scalable molecular simulation. J. Chem. Theor. Comp. *4*, 435-447.

Humphrey, W., Dalke, A., and Schulten, K. (1996). VMD - Visual Molecular Dynamics. J. Molec. Graph. *14*, 33-38.

Hunter, J.D. (2007). Matplotlib: A 2D graphics environment. Comput Sci Eng *9*, 90-95.

Jefferys, E., Sands, Z.A., Shi, J., Sansom, M.S., and Fowler, P.W. (2015). Alchembed: A Computational Method for Incorporating Multiple Proteins into Complex Lipid Geometries. J Chem Theory Comput *11*, 2743-2754.

Marrink, S.J., Risselada, J., Yefimov, S., Tieleman, D.P., and de Vries, A.H. (2007). The MARTINI force field: coarse grained model for biomolecular simulations. J. Phys. Chem. B. *111*, 7812-7824.

McKinney, W. Data Structures for Statistical Computing in Python. In: S. v. d. W. a. J. Millman, editor; 2010. p 51-56.

Michaud-Agrawal, N., Denning, E.J., Woolf, T.B., and Beckstein, O. (2011). MDAnalysis: a toolkit for the analysis of molecular dynamics simulations. J. Comput. Chem. *32*, 2319-2327.

Oliphant, T.E. (2007). Python for scientific computing. Comput Sci Eng *9*, 10-20.

Perera, R., Riley, C., Isaac, G., Hopf-Jannasch, A.S., Moore, R.J., Weitz, K.W., Pasa-Tolic, L., Metz, T.O., Adamec, J., and Kuhn, R.J. (2012). Dengue Virus Infection Perturbs Lipid Homeostasis in Infected Mosquito Cells. Plos Pathog *8*.

Perez, F., and Granger, B.E. (2007). IPython: A system for interactive scientific computing. Comput Sci Eng *9*, 21-29.

Reddy, T., and Rainey, J.K. (2012). Multifaceted substrate capture scheme of a rhomboid protease. J Phys Chem B *116*, 8942-8954.

Reddy, T., Shorthouse, D., Parton, D.L., Jefferys, E., Fowler, P.W., Chavent, M., Baaden, M., and Sansom, M.S. (2015). Nothing to sneeze at: a dynamic and integrative computational model of an influenza A virion. Structure *23*, 584-597.

Schrodinger, LLC. 2010. The PyMOL Molecular Graphics System, Version 1.3r1.

van der Walt, S., Colbert, S.C., and Varoquaux, G. (2011). The NumPy Array: A Structure for Efficient Numerical Computation. Comput Sci Eng *13*, 22-30.

Wadell, H. (1935). Volume, shape, and roundness of quartz particles. J. Geol. *43*, 250–280.

Wolf, M.G., Hoefling, M., Aponte-Santamaría, C., Grubmüller, H., and Groenhof, G. (2010). g_membed: Efficient insertion of a membrane protein into an equilibrated lipid bilayer with minimal perturbation. J. Comput. Chem. *31*, 2169–2174.

Zhang, X.K., Ge, P., Yu, X.K., Brannan, J.M., Bi, G.Q., Zhang, Q.F., Schein, S., and Zhou, Z.H. (2013). Cryo-EM structure of the mature dengue virus at 3.5-angstrom resolution. Nat Struct Mol Biol *20*, 105-U133.
